# The photoacclimation state of stolen chloroplasts affects the light preferences in the photosynthetic sea slug *Elysia crispata*

**DOI:** 10.1242/jeb.251281

**Published:** 2026-02-06

**Authors:** Xochitl G. Vital, Sónia Cruz, Nuno Simões, Paulo Cartaxana, Maite Mascaró

**Affiliations:** ^1^ Posgrado en Ciencias Biológicas, Unidad de Posgrado Edificio D, 1er Piso, Circuito de Posgrados, Ciudad Universitaria, Alcaldía Coyoacan, Ciudad de México C.P. 04510, México; ^2^ UMDI – Sisal, Facultad de Ciencias, Universidad Nacional Autónoma de México, Puerto de Abrigo S/N, Sisal, Yucatan, C.P. 973564, Mexico; ^3^ ECOMARE – Laboratory for Innovation and Sustainability of Marine Biological Resources, CESAM – Centre for Environmental and Marine Studies, Department of Biology, University of Aveiro, Campus Universitário de Santiago, Aveiro 3810-193, Portugal; ^4^ International Chair for Coastal and Marine Studies, Harte Research Institute for Gulf of Mexico Studies, Texas A&M University–Corpus Christi, Corpus Christi, TX 784125, USA; ^5^ Laboratorio Nacional de Resiliencia Costera, Laboratorios Nacionales, CONACYT, C.P. 97356, México

**Keywords:** Behaviour, Kleptoplasty, Mollusca, Phototaxis, Sacoglossa

## Abstract

Photosynthetic sacoglossan sea slugs sequester the chloroplasts of the algae they feed upon and keep these organelles functional in the cells of their ramified digestive system. Whether the stolen chloroplasts – kleptoplasts – influence animal behavioural responses towards light is uncertain. To address this matter, we: (1) determined the light preferences of the photosynthetic sea slug *Elysia crispata* when offered different light spectra (450, 517, 520–650 and 665 nm) and intensities (60, 180, 425 and 1400 µmol photons m^–2^ s^–1^); and (2) established whether the light intensity preferences of *E. crispata* were different when fed algae acclimated to low (40 µmol photons m^–2^ s^–1^) and high irradiance (425 µmol photons m^–2^ s^–1^). Sea slugs were collected from a coral reef in the Gulf of Mexico and transported to the laboratory to perform controlled experiments. During trials, sea slugs exhibited marked exploratory behaviour. However, results show that *E. crispata* avoids red light (665 nm) and prefers low irradiance (60 µmol photons m^–2^ s^–1^), showing that both light spectrum and intensity are relevant to their behaviour. Furthermore, sea slugs increased their selection for high irradiance after being fed algae acclimated to high light. These results support our hypothesis that the acclimation state of the acquired kleptoplasts affects sea slug behaviour towards light. Light perception and photobehaviour in photosynthetic sea slugs seem to depend not only on animal photoreceptors, but also on a communication network between the endosymbiotic chloroplasts and the animal host.

## Teaser

**Highlighted Article:** Light preferences of a photosynthetic sea slug change depending on the photoacclimation of the chloroplasts sequestered from its algal prey.

## INTRODUCTION

Some sea slugs from the superorder Sacoglossa (Mollusca) are the only metazoans capable of long-term maintenance of functional chloroplasts (>1 week and up to several months) from the algae they feed upon, a process known as kleptoplasty (Händeler et al., 2009). Sacoglossan sea slugs feed by puncturing the algae's filaments with a radular tooth and extracting its contents. Specialised cells in the digestive tubules will then phagocytize the chloroplasts (Pierce et al., 2015). These molluscs are an interesting biological model because they can use the photosynthetic products from these ‘stolen’ chloroplasts (kleptoplasts) for their own benefit. Although kleptoplasty in sacoglossan sea slugs has been recognised since the 1960s (e.g. Trench, 1969), much remains to be investigated about the functioning of the endosymbiotic kleptoplasts.

Light is a central resource for photosynthetic organisms as their main energy source, and it also regulates various behavioural and physiological processes in animals (Begon et al., 2006). As photosynthetic animals (Melo Clavijo et al., 2018; Middlebrooks et al., 2011), sea slugs with kleptoplasts possess a unique advantage by diversifying sources of energy supply. However, when exposed to high light intensities, kleptoplasts can accumulate reactive oxygen species (ROS), molecules formed by the addition of electrons to oxygen, causing damage to tissues, thereby compromising their functionality (de Vries et al., 2015; Dorrell and Howe, 2012). Photosymbiotic organisms have negative feedback mechanisms to restore homeostasis, mainly represented by endogenous metabolic and cellular pathways to reduce damage caused by ROS (Dorrell and Howe, 2012). Sea slug species with long-term kleptoplast retention, such as *Elysia timida*, maintain lower ROS levels than short-term retention species, such as *Elysia cornigera* (de Vries et al., 2015). If negative feedback homeostatic mechanisms counteract ROS accumulation in sea slugs, then these animals could be able to detect changes in the photosynthetic efficiency of their kleptoplasts.

In some species of sacoglossan sea slugs, the kleptoplasts retain physiological photoprotective responses under different light intensities, such as changes in pigment concentrations (Cruz et al., 2015; Havurinne and Tyystjärvi, 2020). These changes help dissipate the excessive energy and prevent damage that would inhibit photosynthesis, thereby playing a crucial role in the duration of the photosynthetic activity of kleptoplasts (Cruz and Cartaxana, 2022). The physiological state in which chloroplasts are adjusted to a specific light intensity is called light acclimation or photoacclimation. This acclimation balances the sufficient energy acquisition for metabolic and anabolic processes and protection against excessive energy. Because chloroplasts are the most important light receptors for photosynthesis, acclimation occurs by adjusting composition and function in response to changes in light conditions within them (Bielczynski et al., 2016).

Sea slugs that retain functional kleptoplasts exhibit positive phototaxis (movements directed towards light), unlike species with non-functional retention (Miyamoto et al., 2015; Rahat and Monselise, 1979). Weaver and Clark (1981) found that species with functional kleptoplasts oriented towards light, whereas aposymbiotic species avoided light altogether. Additionally, the behaviour of long-term retention species can be modulated by the acclimation state of their kleptoplasts. In *Elysia viridis*, high-light-acclimated specimens spent significantly more time in high light than low-light-acclimated conspecifics (Cartaxana et al., 2018). Furthermore, shielding of the kleptoplasts from excessive light through the closure of the parapodia occurred at higher irradiance in high-light-acclimated *E. viridis* (Cartaxana et al., 2018).

The sacoglossan sea slug *Elysia crispata*, common in the western Atlantic (Krug et al., 2016), is a long-term retention species because it can maintain functional kleptoplasts for almost 3 months (Curtis et al., 2006). Taylor (1971) reported 500 µmol photons m^–2^ s^–1^ as the optimal light intensity for the kleptoplasts of *E. crispata* to perform photosynthesis. Experiments were conducted at 20°C, an uncommon temperature in the tropical waters *E. crispata* inhabits. Because temperature is a key regulator of metabolism, these results need to be confirmed. Other experiments showed that *E. crispata* chooses light intensities of 200–300 µmol photons m^–2^ s^–1^ over those of 500–600 µmol photons m^–2^ s^–1^, as well as yellow and green light spectra (Weaver and Clark, 1981). However, the sea slugs used in that study had been cultivated in the laboratory, and had no previous exposure to the species' natural environment. Furthermore, behavioural trials were conducted by placing all the specimens together, an experimental setup that has been criticised for not precluding interference between individuals (Martin and Bateson, 2007).

The aims of the present study were to: (1) determine the preferences of *E. crispata* when offered different light spectra (450, 517, 520–650 and 665 nm) and intensities (60, 180, 425 and 1400 µmol photons m^–2^ s^–1^); and (2) establish whether the light intensity preferences of *E. crispata* are modified when fed algae were previously acclimated to high irradiance (425 µmol photons m^–2^ s^–1^). If the behaviour of *E. crispata* is mediated by feedback mechanisms associated with the photosynthetic activity of kleptoplasts, sea slugs are expected to prefer the light intensity to which the kleptoplasts were previously acclimated. Exploring the light preferences of *E. crispata* collected from different native populations through a suitable experimental design will provide a better understanding of the influence of sequestered organelles on their behaviour and distribution.

## MATERIALS AND METHODS

### Collection and maintenance of organisms

*Elysia crispata* (Mörch 1863) was manually collected from reefs at Mahahual, Quintana Roo, Mexico (18°41′10.62″N, 87°42′59.30″W), in November 2019 and Verde in the Sistema Arrecifal Veracruzano, Veracruz, Mexico (19°12′8.80″N, 96°4′17.20″W), in September 2020 and August 2021 at a depth of 3–5 m. Collection was conducted under permits granted by SAGARPA (PPF/DGOPA-082/19 and PPF/DGOPA-061/21) and was limited to the minimum number of organisms required. The individuals had the characteristics of the ‘crispata’ ecotype described by Krug et al. (2016) and measured around 30–50 mm. Upon collection, animals were kept for <48 h in containers with seawater, no food and constant aeration (26°C, ∼50 µmol photons m^–2^ s^–1^) until their transportation to the laboratory (UMDI – Sisal, Facultad de Ciencias, Universidad Nacional Autoónoma de México), where experiments were conducted.

Once at the laboratory, all sea slugs were kept in groups of up to 20 individuals in 14 l maintenance aquaria with recirculating filtered seawater at 26°C and 35 psu, following protocols previously described (Dionísio et al., 2013). The photoperiod was maintained at 12 h:12 h light:dark, reflecting the average conditions of the study area throughout the year. Two Radion XR15W G3 lamps (EcoTech Marine^®^) were used to maintain light at an intensity of ∼100 µmol photons m^–2^ s^–1^ at the water surface (Weaver and Clark, 1981). The total distance from the light source to the bottom of the aquarium was 46 cm, as the lamps were positioned 22 cm above the aquariums with a height of 24 cm. Animals were starved for 24 h and then fed the green macroalgae *Bryopsis pennata* for at least 1 week prior to experiments. This ensured that most kleptoplasts retained by sea slugs came from the same food source. The algae was collected from Sisal, Yucatan, Mexico, and subsequently cultivated in the laboratory at 40 or 425 µmol photons m^–2^ s^–1^ with f/2 Guillard culture medium (Proline^®^; Andersen, 2005). The algae were renewed daily to ensure that the animals consumed the algae acclimated to the light of interest.

### Experimental design

Three experiments were conducted to determine the light preference of *E. crispata* (Table 1; Table S1). Experiment 1 examined whether sea slugs exhibited a preference for light of any of the following colours/spectra: blue (∼450 nm), green (∼517 nm), yellow (∼520–650 nm) or red (∼665 nm). Experiment 2 tested whether these animals showed a preference for any of the four light intensities offered: 60, 180, 425 or 1400 µmol photons m^–2^ s^–1^. In experiments 1 and 2, sea slugs had been fed algae acclimated to low light (40 µmol photons m^–2^ s^–1^). Finally, experiment 3 involved evaluating the preference of *E. crispata* for high (425 µmol photons m^–2^ s^–1^) or low light intensity (60 µmol photons m^–2^ s^–1^) after being fed algae acclimated to high light (425 µmol photons m^–2^ s^–1^). Sea slugs participating in this last experiment were deprived of food for 1 week and then fed *B. pennata* that had been cultivated for 3 weeks at high light intensity (425 µmol photons m^–2^ s^–1^). The data obtained from experiment 3 were compared with that of sea slugs at the corresponding light intensities in experiment 2 (Table 1).

**Table 1.** Light conditions and lamp configuration in the light preference experiments in *Elysia crispata*, as well as the light acclimation of the algae (*Bryopsis pennata*) that the sea slugs consumed

			Treatment (*E. crispata*)			
	Light acclimation (*B. pennata*)		Experimental (CH) lights on		Control (NCH) lights off	
Experiment	λ (nm)	Intensity (µmol photons m^–2^ s^–1^)	λ (nm)	Intensity (µmol photons m^–2^ s^–1^)	λ (nm)	Intensity (µmol photons m^–2^ s^–1^)
						
1	450–525	40	B: 450	∼29	I	–
			G: 517	∼29	II	
			Y: 520–650	∼29	III	
			R: 665	∼29	IV	
						
2	450–525	40	450–475	LL: 60	–	I
			450–475	ML: 180		II
			450–475	HL: 425		III
			450	HH: 1400		IV
						
3	450–475	40	450–475	LL: 60	–	I
			450–475	HL:425		II
	450–475	425	450–475	LL: 60	–	I
			450–475	HL: 425		II
						

CH (choice) represents the light conditions of the chambers when the lights were on; NCH (no choice) represents the same chambers with the lights off. To ensure statistical and behavioural independence, the NCH treatment results from experiments 1 and 2 were used as the control for experiments 2 and 1, respectively. λ, wavelength; B, blue; G, green; Y, yellow; R, red; LL, low light; ML, medium-low light; HL, high light; HH, very high light. I, II, III and IV represent uniform conditions of wavelength and intensities.

**Fig. 1.**
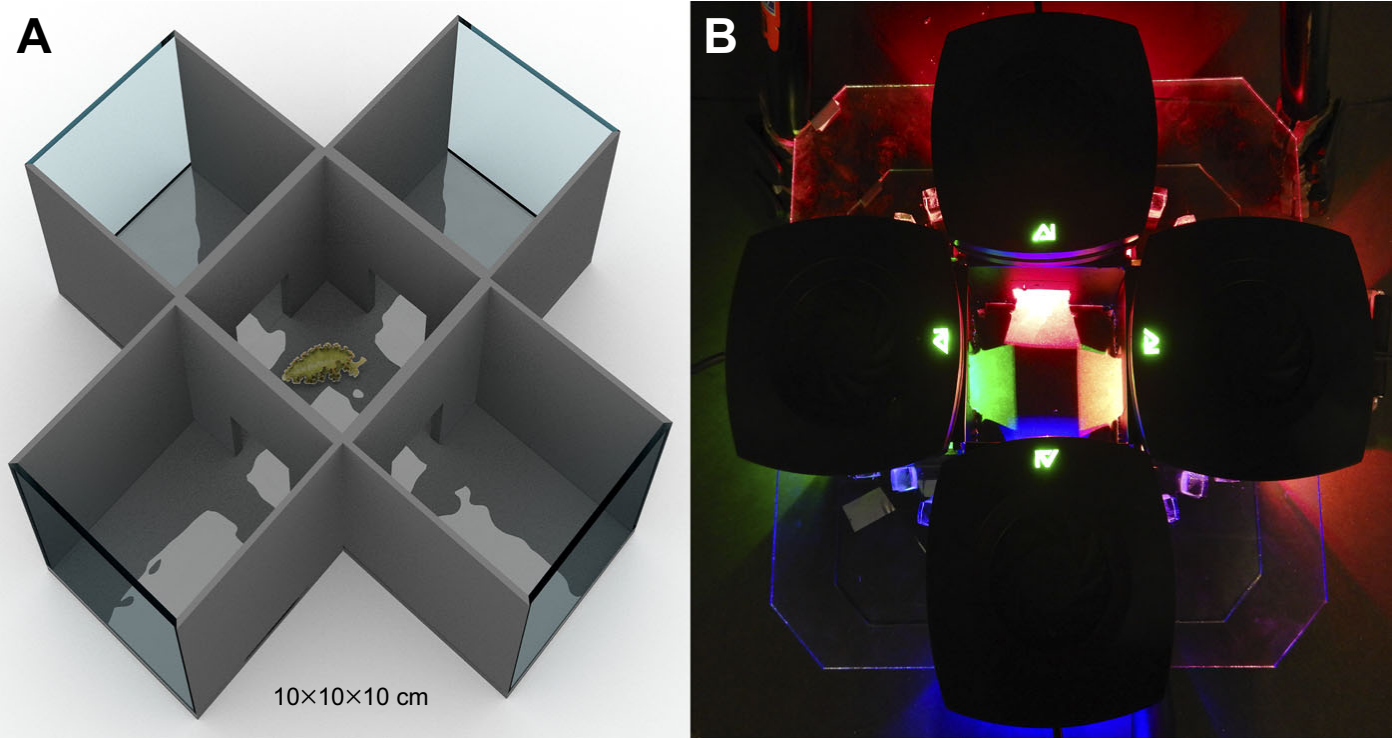
**Experimental device used in light preference trials of *Elysia crispata*.** (A) Cross-sectional view of a digital model of the glass container with dark walls (except on the lateral borders) and a sea slug located in the start position of the trial. (B) Top view of the device with the lights turned on. The lamps were located on top of the transparent acrylic cover.

Light preference was determined using a cross-shaped glass device, with four arms of 10×10×10 cm converging at a central chamber which served as a common compartment (Fig. 1A). To prevent light contamination between chambers, the exterior walls of the device were painted black, and the internal separations were reinforced with thick detachable black plastic. In experiments 1 and 2, access to all four chambers from the centre of the device was kept open, whereas in experiment 3, this access was only kept open for two opposing chambers. The device contained seawater from the maintenance aquarium at a depth of 1 cm (500 ml). This amount of water prevented sea slugs from turning with the ventral side facing the surface and restrained them to the bottom of the device with their dorsal side exposed to the lamps above. A low and constant water depth also avoided bias both in the crawling behaviour of sea slugs and the amount of light reaching their dorsal side, where kleptoplasts are at the highest concentration.

To determine the preference (active selection) for light colour and intensity and distinguish it from passive association (Mascaró et al., 2012), each experiment was conducted by exposing the organisms to two treatments: a choice treatment (CH), where sea slugs were offered different options of light spectra or intensity (accomplished by turning the lamps on); and a no choice treatment (NCH), where sea slugs were offered the same chambers but with the lamps turned off. The latter corresponded to an effective control treatment, as any difference in the behaviour of sea slugs between the CH and NCH conditions would be exclusively attributable to an active selection (Liszka and Underwood, 1990; Mascaró et al., 2012). Thus, preference would be demonstrated if sea slugs choose an alternative in a higher proportion when presented with various options simultaneously, compared with the proportion they choose when there is no choice (Jackson and Underwood, 2007; Underwood et al., 2004). Note that with this experimental design, it was feasible to distinguish a preference or selective behaviour in favour of one or more of the light conditions, or alternatively, a rejection or selective behaviour against any of the alternatives offered.

The experimental device had a transparent acrylic cover to accommodate the lamps (Ai PRIME 16HD^®^; Fig. 1B). A 6 cm diameter opening in the centre of each of the four chambers allowed full passing of light. During the lights-off treatments (NCH), ambient light was kept minimal and consistent across all experiments (0–0.05 µmol photons m^–2^ s^–1^). Because the light spectrum was the variable of interest in experiment 1, the intensity was maintained at ∼2200 lux (∼30 µmol photons m^–2^ s^–1^) in all chambers with lights on (CH). As the variable of interest in experiments 2 and 3 was light intensity, the spectrum of this factor was similar in the CH condition (Table 1). The light intensities and spectra were defined considering previous reports by Taylor (1971), Weaver and Clark (1981) and Vital et al. (2023). Light intensity was measured with a HOBO Pendant^®^ Mx2202 environmental data logger (±10%), and light spectrum with an Ocean Optics^®^ USB4000 fibre optic spectrometer. Light intensity was expressed in µmol photons m^–2^ s^–1^ according to Thimijan and Heins (1983) to enable comparison with the literature on the subject.

### Experimental procedure

**Fig. 2.**
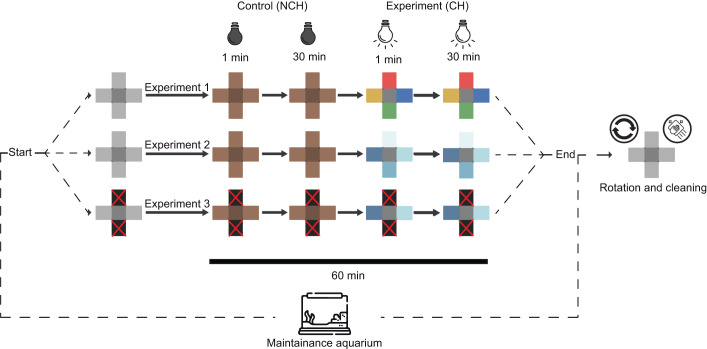
**Diagram of the experimental trials to test the light preference of *E. crispata*.** In each trial of every experiment, one organism from the maintenance aquarium was placed at the centre of the device, then the lid was removed, and its behaviour was observed for 30 min with the lights turned off (NCH, control treatment); afterwards, this procedure was repeated with the lights turned on (CH, experimental treatment). Total duration of a complete trial was 60 min. By the end of each trial, the experimental device was cleaned and rotated, as well as the lamps.

Each trial involved randomly selecting an individual from the maintenance aquaria and placing it in the centre of the experimental device, protected by a PVC lid that prevented the perception of light conditions. After 1 min given for the individual to habituate to the device, the lid was lifted and the trial began (Fig. 2). For 30 min, the sea slug was allowed to explore the chambers with the lights off (NCH treatment). Immediately after, the sea slug was placed back in the centre of the device and the procedure was repeated, but with the lights on (CH treatment; Fig. 2). At the end of each trial, the device was carefully cleaned with freshwater and rinsed with filtered seawater to remove traces of mucus. It was refilled with seawater from the maintenance system and rotated randomly, together with the corresponding lamps.

The trial duration was determined based on previous studies on the behaviour of photosynthetic sea slugs (Cartaxana et al., 2018; Miyamoto et al., 2015), as well as observations during preliminary tests. During CH and NCH treatments of all experiments, a single observer recorded the following variables: (1) first choice frequency: the first chamber in which a sea slug entered at the beginning of each observation, which in turn served to calculate the frequency in which each chamber was first chosen; (2) latency of the first choice: the time (min) elapsed from the start of the trial to the moment in which the sea slug entered the chamber of first choice; (3) duration: the total time (min) a sea slug spent inside each of the four chambers; (4) sampling frequency: the sequence in which a sea slug entered the chambers, which in turn served to calculate the proportion of times a sea slug exited a chamber once it had entered it; and (5) final choice frequency: the chamber in which the sea slug was located at the end of the 30 min period, which in turn served to calculate the frequency in which each chamber was finally chosen (sea slugs found at the central chamber were eliminated from subsequent analyses).

All trials with lights on (CH) were recorded with GoPro Hero 4 and Canon PowerShot G16 cameras to register behavioural variables accurately. In trials with lights off (NCH), only the apical view of the central chamber was recorded with the Canon camera. To prevent any external light bias, a blackout fabric was placed over the experimental device and cameras during NCH trials. Despite the limited amount of light reaching the device, it was possible to distinguish with the naked eye whether the sea slugs entered or exited the chambers. At the end of each trial, all individuals were returned to the maintenance system in a separate aquarium (Fig. 2), except for those used for pigment analysis (*n*=15) in experiment 3. Each individual sea slug was used in only one trial with a total duration of 1 h, and all trials were conducted between 09:00 and 13:30 h.

### Pigment analysis

Analysis of photosynthetic pigments was used to confirm the difference in the acclimation state between algae cultured at different light conditions and sea slugs fed on each type of algae. The pigment composition of 10 sea slugs fed algae acclimated to high light intensity, five sea slugs fed to algae acclimated to low light intensity, along with two samples of respective algae was analysed to confirm whether the light acclimation of the kleptoplasts matched the respective algae profile (Cartaxana et al., 2018; Giossi et al., 2021). Samples were frozen in liquid nitrogen immediately after experiment 3 and freeze-dried. After extraction, photosynthetic pigments were identified and quantified by high performance liquid chromatography as specified in Cruz et al. (2014).

### Statistical analysis

To ensure compliance with the requirement of statistical independence, the data recorded in the NCH treatment in experiment 1 were used as the control for experiment 2, and vice versa. In experiment 3, half of the individuals were randomly labelled as one of the two treatments. The total number of sea slugs involved in the frequency analyses in each of the statistical tests of experiments 1 to 3 is shown in Table S1. The imperfect matching of these numbers was due to irregular outcomes, such as sea slugs found in the central chamber at the end of the trial.

First choice frequency and final choice frequency variables in experiments 1 and 2 were analysed with *G*-tests (likelihood ratio) in two-dimensional contingency tables (Liszka and Underwood, 1990; Zar, 2009), with NCH and CH treatments as the first dimension and the four light options (either colour or intensity) as the second. These behavioural variables in experiment 3 were analysed with *G*-tests in three-dimensional contingency tables, with NCH and CH treatments, the two light intensity options (high: 425 µmol photons m^–2^ s^–1^; low: 60 µmol photons m^–2^ s^–1^) and the low or high light intensity to which the algae had been acclimated as the first, second and third dimensions, respectively.

The sampling frequency in experiments 1 and 2 was analysed with three-dimensional contingency tables with NCH and CH treatments as the first dimension, the four light options as the second, and the number of times sea slugs exited or remained in a chamber once they had entered it as the third. Note that because a sea slug could enter any chamber more than once in a single trial, the total frequency did not correspond to the number of sea slugs involved in each experiment (Table S1). Sampling frequency in experiment 3 would have needed a four-dimensional contingency table; however, owing to an excessively low number of sea slugs, no test of hypothesis was applied, and results were only plotted and described.

Latency and duration in experiments 1 and 2 were analysed by comparing differences in NCH and CH treatments for each spectrum or light intensity option using Mann–Whitney (M–W) *U*-tests (16 in total). These variables in experiment 3 were analysed with M–W tests (four in total) applied per variable (duration and latency) and for each option offered (LL and HL). Finally, a M–W test was used to analyse the *trans*-neoxanthin/chlorophyll *a* pigment ratio to assess the differences in sea slug acclimation.

The graphs and analyses were conducted in Tableau 2021.1 and R v. 3.5.3 (https://www.r-project.org/), using the *ggplot* 3.3.5 (Wickham, 2016) and *stats* 3.5.3 packages, or in spreadsheets according to the formulas in Zar (2009). Results were considered statistically significant at *P*<0.05.

## RESULTS

Despite the highly variable conduct towards light in the 115 *E. crispata* examined, evident patterns were noted after collectively considering all five behavioural variables. In all three experiments, both the first choice of sea slugs and the latency of first choice in treatments with the lights turned on (CH) were statistically similar to those with the lights turned off (NCH; Table S2), suggesting that the response of sea slugs to an initial stimulus of either light intensity or spectra was not the result of an active selection. By contrast, statistical analyses of the duration, sampling frequency and final choice of sea slugs of the light options offered under CH and NCH treatments yielded results that varied from one experiment to another (Table S2), as described below.

### Preference of light spectrum

**Fig. 3.**
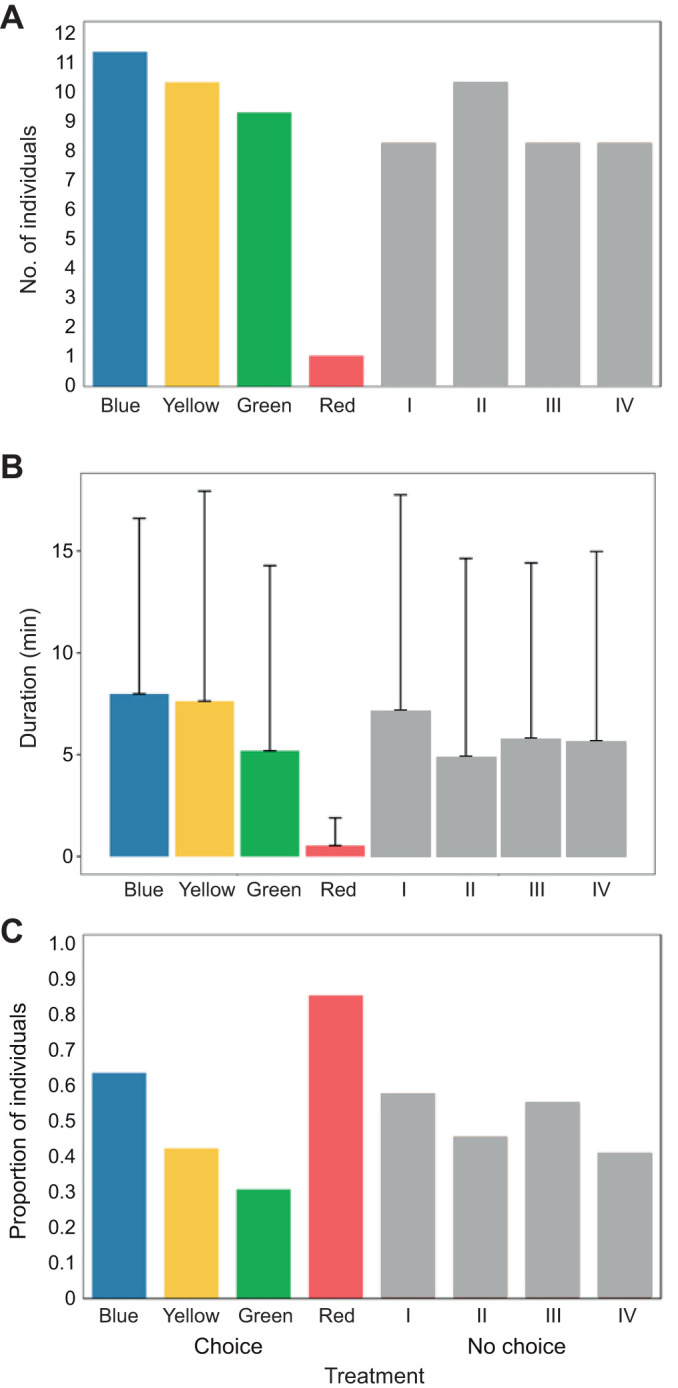
**Light spectrum preference of *E. crispata* (experiment 1).** (A) Final choice frequency, (B) duration (means±s.d.) and (C) sampling frequency. Lights of different colours were either turned on (CH) or off (NCH); numbers I–IV correspond to chambers without light (control; see Table 1). See Table S1 for sample sizes.

In experiment 1, most *E. crispata* selected chambers with light colours other than red as their final choice (Fig. 3A; Table S3). However, no significant differences were found between the control conditions (NCH) and those where sea slugs could choose different spectra (CH).

When lights were turned on, *E. crispata* spent more time in the yellow, blue and green lights compared with time spent in the same chambers without light (Fig. 3B; Movie 1). Although duration was highly variable, M–W tests showed sea slugs spent significantly less time in the red light under CH than under NCH conditions.

When offered a choice, most sea slugs (58%) inspected at least two chambers with different colours. Sea slugs entered the chamber with the blue spectrum most frequently, making it the most explored. By contrast, five out of six animals that entered the red light left before the 30 min trial period ended, making it the spectrum most frequently abandoned after being explored (Fig. 3C). Despite this trend, the *G*-test found no significant differences in sampling frequency between NCH and CH treatments.

Overall results of experiment 1 showed that sea slugs did not exhibit a preference or active selection for blue, yellow or green light (Table 2). However, the red colour was generally avoided by *E. crispata* throughout the trials.

**Table 2.** Results and brief interpretation of the analyses performed on each variable in the three preference experiments of *E. crispata*

Response variable	Statistical test	Statistical significance	Interpretation
Experiment 1: light spectrum			
Final choice frequency	2D *G*-test	n.s.	Light colour did not change the frequency of the final choice of chamber
Duration	M–W	*Red	Light colour significantly decreased the duration in the chamber with red light
Sampling frequency	3D *G*-test	n.s.	Light colour did not change the proportion of exits of the total entries in each chamber
First choice frequency	2D *G*-test	n.s.	Light colour did not change the frequency of the first choice of chamber
Latency	M–W	*Green	Light colour significantly decreased the latency to enter the chamber with green light
Experiment 2: light intensity			
Final choice frequency	2D *G*-test	*	Light intensity significantly changed the frequency of the final choice of chamber
Duration	M–W	*60	Light intensity significantly increased the duration in the chamber with 60 µmol photons m^–2^ s^–1^
Sampling frequency	3D *G*-test	*	Light intensity significantly changed the proportion of exits of the total entries in each chamber
First choice frequency	2D *G*-test	n.s.	Light intensity did not change the frequency of the first choice of chamber
Latency	M–W	n.s.	Light intensity did not change the latency to enter the first choice of chamber
Experiment 3: light acclimation			
Final choice frequency	3D *G*-test	*	Light acclimation significantly changed the frequency of the final choice of chamber with contrasting light intensities
Duration	M–W	n.s.	Light acclimation did not change the duration in chambers with contrasting light intensity
Sampling frequency	Not analysed	–	
First choice frequency	3D *G*-test	n.s.	Light acclimation did not change the frequency of the first choice of chamber with contrasting light intensities
Latency	M–W	n.s.	Light acclimation did not change the latency to enter the first choice of chamber with contrasting light intensity

M–W, Mann–Whitney; n.s., not significant. *P<0.05.

### Preference of light intensity

When offered a choice in experiment 2, the final frequency of sea slugs in chambers with low and medium-low intensity was almost six times higher than in high and very high light intensities. By contrast, when lights were turned off, sea slugs were found in similar numbers in all chambers. Differences in the frequency of sea slugs under CH and NCH treatments were significant, suggesting the preference for low light intensities (and the rejection of high light intensities) was the result of active selection.

**Fig. 4.**
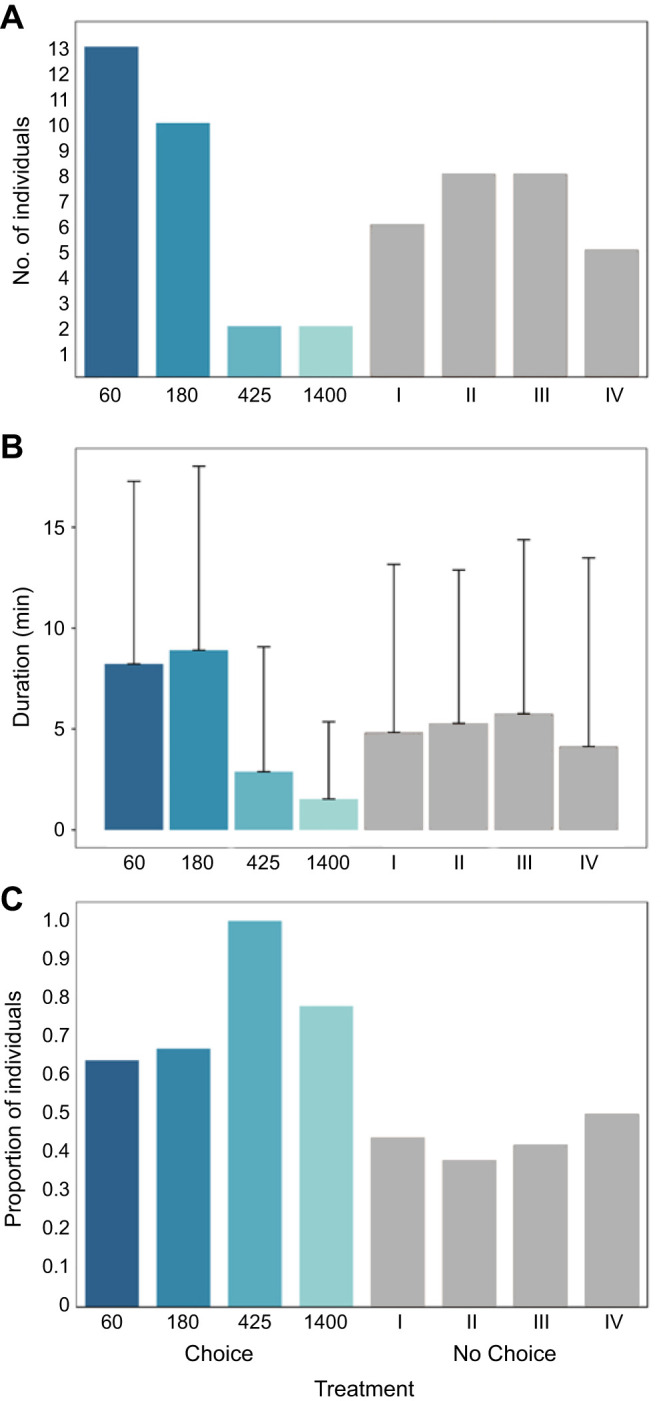
**Light intensity preference of *E. crispata* (experiment 2).** (A) Final choice frequency, (B) duration (means±s.d.) and (C) sampling frequency. Lights of different intensities were either turned on (CH) or off (NCH); numbers I–IV correspond to chambers without light (control; see Table 1). See Table S1 for sample sizes.

When given an option, sea slugs spent more time in chambers with the lowest light intensities (Fig. 4B; Movie 2), but only one of the M–W tests comparing CH and NCH treatments was statistically significant (60 µmol photons m^–2^ s^–1^; Table S2).

Sampling frequency in experiment 2 showed that almost 60% of sea slugs explored more than two options of light intensity when given a choice. Among the sea slugs that entered chambers with different light intensities under CH conditions, a greater proportion left the high and very high-light intensity chambers compared with low and medium-low light intensity (Fig. 4C). This behaviour was significantly different from that displayed when sea slugs had no choice.

Overall, results of experiment 2 indicate that *E. crispata* actively selected lower light intensities (60–180 µmol photons m^–2^ s^–1^) over higher irradiances (425–1400 µmol photons m^–2^ s^–1^). Such indication stems principally from the way sea slugs explored the available light intensities, the time spent in each of them, and the final frequency in which sea slugs were found in the corresponding chambers (Table 2).

### Light acclimation-dependent preference

The *trans*-neoxanthin/chlorophyll *a* pigment ratio in sea slugs previously fed *B. pennata* acclimated to HL was significantly higher (*U*=50, *P*<0.05) in both algae and sea slugs (0.379±0.027 and 0.462±0.192) compared with algae cultivated in low light (LL) and the sea slugs that consumed them (0.041±0.004 and 0.107±0.058, respectively; Table S5).

**Fig. 5.**
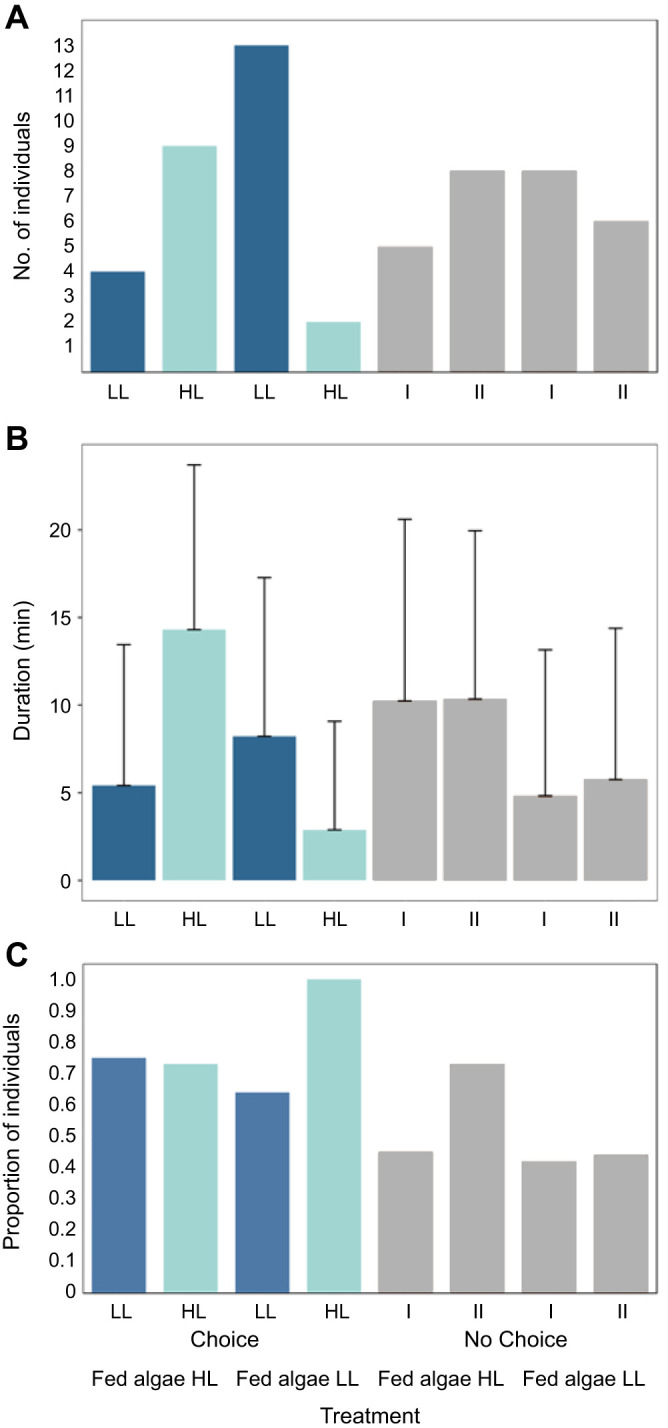
**Light intensity preference of *E. crispata* fed with algae acclimated to either 40 or 425 µmol photons m^–2^ s^–1^ [low light (LL) and high light (HL), respectively] (experiment 3).** (A) Final choice frequency, (B) duration (means±s.d.) and (C) sampling frequency. Lights of different intensities were either turned on (CH) or off (NCH); numbers I–IV correspond to chambers without light (control; see Table 1). See Table S1 for sample sizes.

Sea slugs fed with algae acclimated to HL were more often observed in HL chambers compared with those fed with algae acclimated to LL (Fig. 5A; Movie 3). Moreover, the final frequency of sea slugs in HL and LL chambers under CH treatments differed significantly from those in NCH, indicating that the association between sea slug preference for light intensity and algae acclimation is the result of an active selection.

In contrast to the results of experiment 2, *E. crispata* fed with algae acclimated to HL almost triplicated the time spent in HL compared to LL chambers when offered a choice. By contrast, the average duration of sea slugs in all chambers in NCH treatments was very similar (Fig. 5B). Despite this trend, the durations between NCH and CH treatments in experiment 3 were statistically similar.

Sea slugs in experiment 3 also explored the light intensity options available, but those fed with algae acclimated to low light intensity exited the HL chamber in a higher proportion than they did the LL chamber when given a choice. In contrast, when sea slugs were fed with algae acclimated to high light, the proportion of exits from either type of light was similar (Fig. 5C). Although differences between CH and NCH treatments were not statistically confirmed, sea slugs in experiment 3 stayed in HL chambers once they had entered more frequently compared with sea slugs in experiment 2.

Overall, results of experiment 3 showed that *E. crispata* modifies its choice for light intensity after consuming algae acclimated to high light. Based on the final choice frequency of sea slugs in the different chambers alone, this behavioural change is the result of an active selection (Table 2).

## DISCUSSION

### *Elysia crispata* passively avoids red light

Results of experiments on the preference for colour suggest that *E. crispata* might passively circumvent red light rather than actively reject it, because wavelengths of ∼650 nm do not constitute an equally viable spectral alternative. Long wavelengths corresponding to red light (∼650 nm) are absorbed in the ocean at shallow depths, with only 1% reaching 10 m in translucent waters (Lalli and Parsons, 1997). Despite experimental organisms being collected from locations where long wavelengths were part of the natural light spectra (3–5 m depth), it is possible red light constituted a cue triggering the movement of sea slugs to explore other light qualities within the experimental device. Evidence supporting this idea includes the high frequency at which sea slugs exited the red chamber after having entered it relative to other colour alternatives, together with the overall less time spent in the red-light chamber.

To perceive light cues, sacoglossan sea slugs must possess photoreceptors capable of detecting different wavelengths, which they can recognise owing to the absorption properties of photoreceptor pigments (Stavenga, 2010). Molluscs have two photopigment-forming proteins: cryptochromes, restricted to blue-light sensitivity (370–440 nm), and opsins, which exhibit a wide range of maximum absorbance wavelengths (McElroy et al., 2023). The visual opsin found in gastropods, rhodopsin, absorbs maximally in the blue–green range (450–550 nm; Stavenga, 2010). Genes encoding six opsins and five cryptochromes have been found in sacoglossan species (McElroy et al., 2023), demonstrating broad wavelength detection capacity within this taxonomic group.

Red light is the optimal spectrum for green algae photosystems (Butler, 1978). However, evidence suggests that sacoglossans may be unable to detect red light despite its importance for their photosynthetic symbionts. Photoreceptors in the eyes of *Elysia timida* distinguish appropriate from excessive light intensities and are partially responsible for the opening and closing of parapodia, thereby controlling the light reaching the kleptoplasts (Rahat and Monselise, 1979). Rahat and Monselise (1979) found that eyeless individuals perceived only 540 nm (green) light, showing no parapodial response to red (640 nm) light. This limitation is not unique to sacoglossans; the photosymbiotic flatworm *Symsagittifera roscoffensis* also does not react to red light, possibly owing to an inability to perceive it (Nissen et al., 2015). Thus, it is possible that sacoglossan sea slugs rely on their rhodopsin-containing photoreceptors that function optimally in the blue–green range, rather than detecting wavelengths that could directly benefit their kleptoplasts.

Twelve photosynthetic pigments with maximum absorption in 414–480 nm were found in *E. crispata* from the same location of the present study (Vital et al., 2021). The main carotenoids were siphonoxanthin and siphonein, pigments from macroalgal food sources that are particularly efficient at absorbing green (510–550 nm) and blue–green light (470–510 nm) (Marques et al., 2021; Wang et al., 2013). Previous experiments found that *E. crispata* selected yellow light, with red light being the least preferred (Weaver and Clark, 1981), thus partially coinciding with our results. If the photoreceptors in *E. crispata*, including those from kleptoplasts, have pigments with maximum absorption in the blue and green spectra, sea slugs possibly perceived the red-light chamber as one with the absence of light altogether, hence explaining its avoidance.

### *Elysia crispata* prefers low light intensities

In experiment 2, *E. crispata* preferred 60 µmol photons m^–2^ s^–1^, a light intensity much lower than previously reported (200–280 µmol photons m^–2^ s^–1^; Weaver and Clark, 1981; Burgués Palau et al., 2024). These previous studies used cultivated sea slugs or specimens collected directly from natural habitats and exposed them to 0 and 1396 µmol photons m^–2^ s^–1^. Despite procedural differences, *E. crispata* consistently chose intensities below the mean value measured at their natural habitats. Caribbean coral reefs receive up to 2500 µmol photons m^–2^ s^–1^ at the surface (Vermeij and Bak, 2002). Burgués Palau et al. (2024) collected specimens from shallow Curaçao reefs, where mean light intensity is 652.02±397.01 µmol photons m^–2^ s^–1^. By contrast, our sea slugs came from southern Gulf of Mexico reefs, where the maximum and mean intensity at the zenith was 1260 and 245 µmol photons m^–2^ s^–1^, respectively (Vital et al., 2023). Furthermore, the preferred light intensity observed in our study was either less than 30% (60 µmol photons m^–2^ s^–1^ in experiment 2) or, at most, similar (425 µmol photons m^–2^ s^–1^ in experiment 3) to the optimal range for chloroplast photosynthesis (225 to 500 µmol photons m^–2^ s^–1^; Burgués Palau et al., 2024; Taylor, 1971).

The selection of low light intensities by this and other *Elysia* species has been associated with a self-protective strategy to mitigate damage caused by excessive light to the retained kleptoplasts (Jesus et al., 2010; Vieira et al., 2009). When exposed to high light intensities, highly toxic ROS formed within the kleptoplasts may extend their damaging effects beyond this organelle, affecting the membranes of animal cells. If oxidative stress surpasses a certain threshold, it may act as a biochemical signal, prompting the sea slug to exhibit behaviours that protect the kleptoplasts from further light exposure, such as moving in search of a less stressful environment or activating parapodial shading (Burgués Palau et al., 2024).

Indeed, the relationship between oxidative stress and various adaptive behaviours in marine animals is well documented and has been closely linked to changes in physiological demands from environmental challenges (e.g. Menon et al., 2023; Snitman et al., 2022). For example, short-term exposure to hydrogen peroxide in bivalves induces changes in corticosteroid metabolism, likely influencing withdrawal behaviours or reducing responsiveness to stimuli (Hallmann et al., 2025). Much of this research suggests that oxidative stress modifies behavioural responses by altering neuroendocrine regulation (Grădinariu et al., 2025) or decreasing energy availability through mitochondrial damage (Meza-Buendía et al., 2021). Comparing starvation and oxidative stress in *Elysia cornigera* and *E. timida* revealed effective cellular signalling, involving increased superoxide concentrations, detection of damaged organelles, upregulation of genes encoding ROS scavengers, and initiation of autophagosomal activity (de Vries et al., 2015). Oxidative activity has also been detected in the digestive gland of *E. crispata* under high light exposure; however, the authors could not determine whether ROS production resulted from kleptoplast photodamage or other physiological processes such as mitochondrial respiration (Burgués Palau et al., 2024). Although ROS signalling at the cell level has long been documented (Li et al., 2013), further research is needed to elucidate the specific feedback mechanisms underlying photoprotective behaviour in the symbiotic sacoglossan–plastid relationship.

Maximum absorbance (λ_max_) in blue and green light allows algae to exploit a wide range of wavelengths available in the environment and access a diversity of habitats (Wang et al., 2013). However, how Bryopsidales cope with excessive irradiance remains unclear, as they lack photoprotection mechanisms present in other green algae and plants, such as the xanthophyll cycle and state transitions (Christa et al., 2017; Giossi et al., 2021; Havurinne et al., 2025). Consequently, photosynthetic sea slugs feeding on Bryopsidales may exhibit kleptoplast-shielding behaviours under excess light (Cartaxana et al., 2019). In *E. viridis*, kleptoplast acclimation influenced parapodial opening/closing and light preference (5–80 µmol photons m^–2^ s^–1^) when sea slugs were fed algae cultured at 40 µmol photons m^–2^ s^–1^ (Cartaxana et al., 2018). Furthermore, parapodia closure has been reported to reduce light reaching the kleptoplasts by 200 µmol photons m^–2^ s^–1^ (Burgués Palau et al., 2024). Our experiments showed that *E. crispata* spent more time in chambers with 60 and 180 µmol photons m^–2^ s^–1^, clearly avoiding excessive irradiance. Although we also noted that parapodial closing increased with light intensity, the main photoprotective behaviour displayed herein was to avoid potentially deleterious light conditions.

### *Elysia crispata* shifts its preference for high irradiance after being fed high-light acclimated algae

Interestingly, high light intensities so clearly avoided by *E. crispata* became the most frequently selected by merely altering the light acclimation conditions of the *B. pennata* consumed by sea slugs. The behavioural versatility implied by this shift could be founded on a relatively simple control signal turning on and off the photosynthetic system of the kleptoplasts. Because kleptoplasts maintain the photosynthetic ability but cannot themselves acclimate to light variations (Cartaxana et al., 2018; Serôdio et al., 2014), algal acclimation conditions when consumed by sea slugs should influence the extent to which kleptoplasty is advantageous for the animal. If feedback mechanisms triggering high light avoidance in sea slugs are associated with the limits of the photosynthetic capacity of the kleptoplasts (derived from ROS accumulation through oxidative stress), then plastid acclimation should determine the threshold between beneficial and photodamaging light exposures. Plastids from high-light-acclimated algae might allow host sea slugs to add photosynthetic profit at intensities as high as 500 µmol photons m^–2^ s^–1^ before reaching the threshold that elicits photoprotective avoidance behaviours. This would explain why the *E. crispata* that consumed algae acclimated to 40 µmol photons m^–2^ s^–1^ preferred low light (60 µmol photons m^–2^ s^–1^) but shifted its preference to the high intensity alternative (425 µmol photons m^–2^ s^–1^) after feeding on algae acclimated to 425 µmol photons m^–2^ s^–1^.

Previous studies have shown that species with long-term kleptoplast retention explore more during phototaxis experiments than those with short-term or no retention (Burgués Palau et al., 2024; Cartaxana et al., 2018; Miyamoto et al., 2015; Schmitt and Wägele, 2011). Our results support these findings and point to the active exploratory behaviour displayed by *E. crispata* in which sea slugs sampled the available light conditions by entering, staying and exiting experimental chambers when light options were available. The relatively large standard deviations in latency and duration reflect the variability in exploratory behaviour, and correspond to the spatial and temporal patterns of *E. crispata* in its natural environment (Vital et al., 2023). It would be interesting to evaluate the behaviour of ‘albino’ individuals that do not retain chloroplasts (recorded in *E. crispata* and *E. chlorotica*; Cartaxana et al., 2022; Pierce et al., 2015), as we would expect them not to exhibit such exploratory behaviours.

Studies on light preference in *Elysia* have been performed under varying experimental conditions, including different specimen origins (collection localities, cultured organisms), light acclimation and maintenance conditions (e.g. temperature), and experimental designs with differing ranges of light alternatives. These methodological inconsistencies make results difficult to compare across studies and likely influence findings. Future studies should address these limitations by using the widest possible range of light intensities found in natural environments and including appropriate controls that compare behaviour of the same species with and without the tested signals (Burgués Palau et al., 2024; Cartaxana et al., 2018; Miyamoto et al., 2015; Schmitt and Wägele, 2011). Researchers should also assess the origin of chloroplasts in collected specimens, as different algal species provide kleptoplasts with distinct photoprotection mechanisms (Middlebrooks et al., 2019; table S1 in Vital et al., 2021). Finally, studies should account for the distribution and light regime of source algae, as these conditions define kleptoplast performance and can shape the light preferences of sea slugs.

Conclusions drawn from the present study support that the quantity and quality of available light influence the behaviour of *E. crispata*. Our findings suggest that this photosynthetic sea slug can detect diverse wavelengths and intensities and can modify its preference depending on the photoacclimation state of its kleptoplasts. Monitoring irradiance and detecting spectra in animals is fundamental for circadian regulation, phototaxis and body orientation (Nilsson, 2009), and constitutes an adaptive response to temporal and spatial changes in the aquatic environment (Kirk, 2011). Results herein emphasise the presence of physiological and behavioural traits that may enable *E. crispata* to explore its surrounding light environment within a time scale of a few hours. Such a behavioural feature has been previously regarded as an explanation for daily changes in the bathymetric distribution of this species in its natural habitat (Vital et al., 2023).

## Supplementary Material

10.1242/jexbio.251281_sup1 Supplementary information
