# Supplementary material for: The photoacclimation state of stolen chloroplasts affects the light preferences in the photosynthetic sea slug Elysia crispata
Source: J Exp Biol. 2026 Feb 6;229(3):jeb251281. doi: 10.1242/jeb.251281 (PMC12912266; doi:10.1242/jeb.251281)
Supplement: Supplementary information [file jexbio-229-251281-s1.pdf]

Table S1. Total number (*n*) of sea slugs involved in the frequency analyses in each of the experiments. LL = 40 μmol photons m^{-2} s^{-1}; HL = 425 μmol photons m^{-2} s^{-1}.

Experiment	Algae acclimation	*Final choice Frequency*		*Sampling Frequency*		*First-choice Frequency*	
		CH	NCH	CH	NCH	CH	NCH
1: colour	LL	31	34	54	53	36	36
2: intensity	LL	27	27	65	40	32	29
3: acclimation	LL	15	14	35	21	21	16
	HL	13	13	15	22	13	14

Table S2. Observed (and expected) values of the final choice frequency and first-choice frequency of *Elysia crispata* with the lights on (CH condition) in the three preference experiments. LL = 60 μmol photons m^{-2} s^{-1}; HL = 425 μmol photons m^{-2} s^{-1}. Light intensity treatments in Experiment 2 (parentheses) are in μmol photons m^{-2} s^{-1}.

Response variable	Options			
Experiment 1. Colour	Blue	Yellow	Green	Red
Final choice frequency	11 (2.39)	10 (2.30)	9 (2.19)	1 (0)
First-choice frequency	13 (2.56)	11 (2.39)	8 (2.07)	4 (1.38)
Experiment 2. Intensity	I (60)	II (180)	III (425)	IV (1400)
Final choice frequency	13 (2.56)	10 (2.3)	2 (0.69)	2 (0.69)
First-choice frequency	13 (2.56)	8 (2.079)	8 (2.07)	3 (1.09)
Experiment 3. Acclimation	Fed HL		Fed LL	
	LL	HL	LL	HL
Final choice frequency	4 (1.38)	9 (2.19)	13 (2.56)	2 (0.69)
First-choice frequency	4 (1.38)	9 (2.19)	13 (2.56)	8 (2.07)

Journal of Experimental Biology • Supplementary information

Table S3. Latency and duration of *Elysia crispata* with the lights on (CH condition) in the three preference experiments. Values represent the mean ± standard deviation (s.d.). LL = 60 μmol photons m^{-2} s^{-1}; HL = 425 μmol photons m^{-2} s^{-1}. The units of the variables are minutes.

Response variable		Options		
Experiment 1. Spectrum	Blue	Yellow	Green	Red
Duration	7.98 ± 8.6	7.62 ± 10.3	5.18 ± 9	0.53 ± 1.3
Latency	4.33 ± 5.4	1.68 ± 0.9	1.9 ± 1.4	5.9 ± 5
Experiment 2. Intensity	I (60)	II (180)	III (425)	IV (1400)
Duration	8.22 ± 9	8.9 ± 9.1	2.88 ± 6.1	1.5 ± 3.8
Latency	4.33 ± 3.1	3.62 ± 2.6	2.16 ± 0.8	4.65 ± 5.8
Experiment 3. Acclimation	Fed HL		Fed LL	
	LL	HL	LL	HL
Duration	5.41 ± 8	14.3 ± 9.4	8.22 ± 9	2.88 ± 6.1
Latency	4.83 ± 5.6	3.98 ± 3	4.33 ± 3.1	2.16 ± 0.8

Journal of Experimental Biology • Supplementary information

Table S4. Results of the statistical tests used to assess differences between the treatment with the lights on (CH) and lights off (NCH) in the three preference experiments of *Elysia crispata*. M-W= Mann-Whitney; LL = 60 µmol photons m^{-2} s^{-1}; HL = 425 µmol photons m^{-2} s^{-1}. Light intensity treatments in Experiment 2 (in parentheses) are in µmol photons m^{-2} s^{-1}. Asterisks (*) indicate differences considered to be significant at $p < 0.05$.

Response variable	Statistical parameters			
Experiment 1. Colour				
G-test	*G*	*df*	*p*	
Final choice frequency	6.38	3	0.094	
First-choice frequency	0.94	3	0.82	
Sampling frequency	14.87	10	0.14	
M-W test	Blue	Yellow	Green	Red
Duration	U = 579<br>*p* = 0.412	U = 712<br>*p* = 0.399	U = 522.5<br>*p* = 0.101	U = 803<br>*p* = 0.024*
Latency	U = 104.5<br>*p* = 0.317	U = 74<br>*p* = 0.068	U = 53<br>*p* = 0.03*	U = 5<br>*p* = 0.164
Experiment 2. Intensity				
G-test	*G*	*df*	*p*	
Final choice frequency	8.04	3	0.045*	
First-choice frequency	1.94	3	0.584	
Sampling frequency	22.29	10	0.05*	
M-W test	I (60)	II (180)	III (425)	IV (1400)
Duration	*U* = 314<br>*p* = 0.021*	U = 349<br>*p* = 0.083	U = 508.5<br>*p* = 0.475	U = 449.5<br>*p* = 0.789
Latency	U = 38<br>*p* = 0.578	U = 37<br>*p* = 0.961	U = 40.5<br>*p* = 0.7	U = 3<br>*p* = 0.4
Experiment 3. Acclimation				
G-test	*G*	*df*	*p*	
Final choice frequency	11.97	4	< 0.05*	
First-choice frequency	4.18	4	0.18	
Sampling frequency		Not tested		
M-W test		LL		HL
Duration	U = 119	*p* = 0.167	U = 69	*p* = 0.295
Latency	U = 19	*p* = 0.682	U = 31	*p* = 0.688

Journal of Experimental Biology • Supplementary information

Table S5. Photosynthetic pigment to chlorophyll *a* ratios in *Elysia crispata* after consuming algae acclimated to low light (LL) or high light (HL), and of *Bryopsis pennata* after being cultured in the laboratory in LL or HL for at least three weeks. Values represent the mean ± standard deviation (s.d.). LL = 40 μmol photons m^{-2} s^{-1}; HL = 425 μmol photons m^{-2} s^{-1}.

Pigment / Chl *a*	*E. crispata* LL (n = 5)	*E. crispata* HL (n = 10)	*B. pennata* LL (n = 2)	*B. pennata* HL (n = 2)
Siphonoxantin	0.147 ± 0.116	0.225 ± 0.095	0.265 ± 0.024	0.171 ± 0.059
trans-neoxanthin*	0.107 ± 0.058	0.462 ± 0.192	0.041 ± 0.004	0.379 ± 0.027
cis-neoxanthin	0.278 ± 0.067	0.397 ± 0.102	0.213 ± 0.018	0.219 ± 0.056
Violaxanthin	0.099 ± 0.040	0.139 ± 0.023	0.024 ± 0.004	0.088 ± 0.037
Lutein	0.272 ± 0.217	0.173 ± 0.190	n.d.	0.049 ± 0.017
Zeaxanthin	0.089 ± 0.056	0.075 ± 0.050	n.d.	0.049 ± 0.009
Siphonein	0.102 ± 0.074	0.165 ± 0.054	0.149 ± 0.004	0.095 ± 0.025
Clorophyll *b*	0.816 ± 0.140	0.905 ± 0.078	0.977 ± 0.050	0.807 ± 0.102
βε-Carotene	0.078 ± 0.115	0.099 ± 0.070	0.071 ± 0.001	0.087 ± 0.047
ββ-Carotene	0.020 ± 0.019	0.009 ± 0.013	n.d.	0.061 ± 0.046

Asterisk (*) indicates the pigment that confirms the light acclimation of the chloroplasts.

n.d. – not detected.

Journal of Experimental Biology • Supplementary information

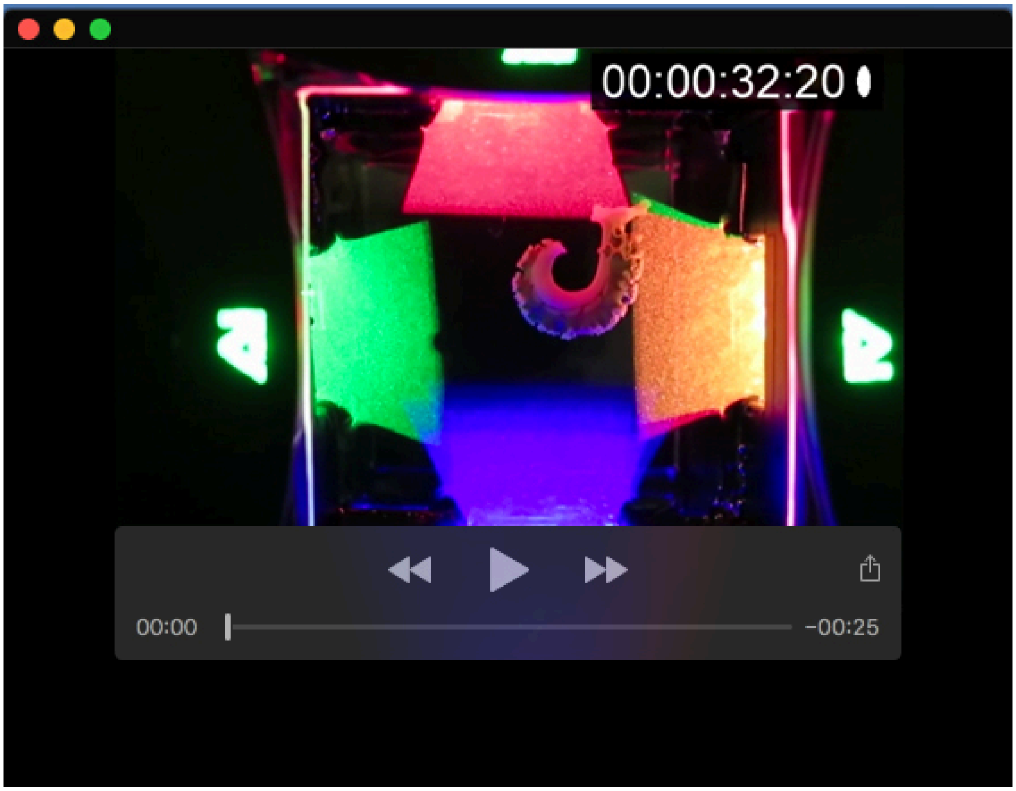

Movie 1. Trial to test the light spectrum preference of *Elysia crispata* (Experiment 1) with the lights on (CH, experimental treatment).

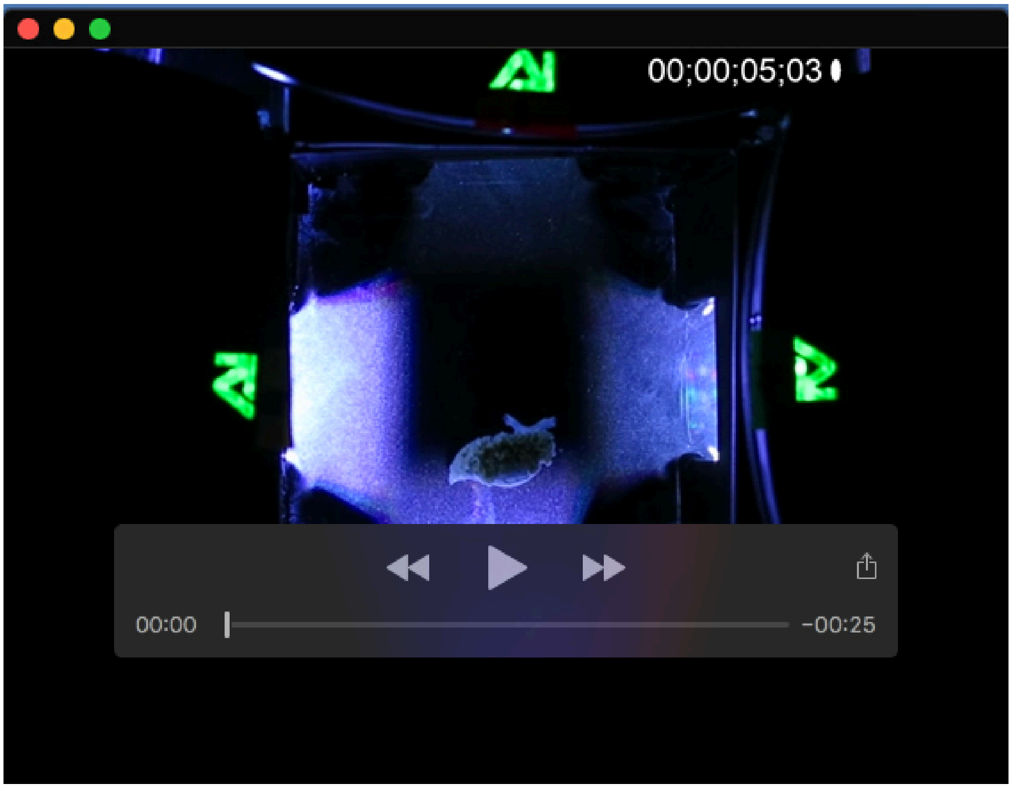

Movie 2. Trial to test the light intensity preference of *Elysia crispata* (Experiment 2) with the lights on (CH, experimental treatment).

Journal of Experimental Biology • Supplementary information

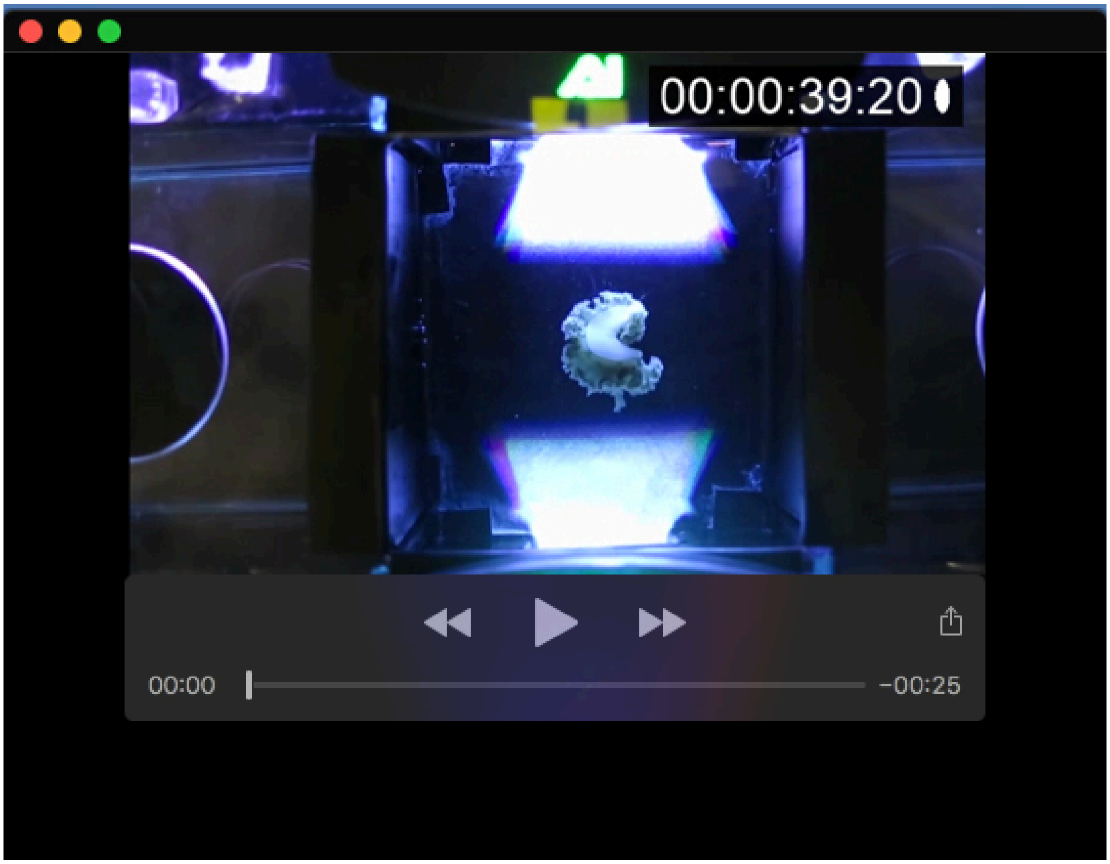

Movie 3. Trial to test the light intensity preference of *Elysia crispata* fed with algae acclimated to 425 µmol photons m^{-2} s^{-1} (Experiment 3) with the lights on (CH, experimental treatment).
